# Transient conduction block of the superior vena cava following pulsed field ablation of the right superior pulmonary vein

**DOI:** 10.1002/joa3.70095

**Published:** 2025-05-15

**Authors:** Kenta Takahashi, Toshio Makita, Shinya Shimoshige, Taishi Kuwahara

**Affiliations:** ^1^ Department of Cardiology Tokyo Heart Rhythm Clinic Tokyo Japan

**Keywords:** atrial fibrillation, catheter ablation, pulsed‐field ablation, right superior pulmonary vein, superior vena cava

## Abstract

This case demonstrates that an immediate superior vena cava (SVC) conduction block may occur during pulsed field ablation (PFA) due to anatomical proximity, a positive tissue proximity index, and a narrow SVC diameter. However, lesions not directly influenced by PFA tend to regress and may be reversible, leading to SVC potential reconduction. CS, coronary sinus; d, distal; LAO, left oblique view; LL, left lateral; p, proximal; PA, posteroanterior view; RA, right atrial; RAO, right oblique view; RSPV, right superior pulmonary vein; RV, right ventricular; SUP, superior view.

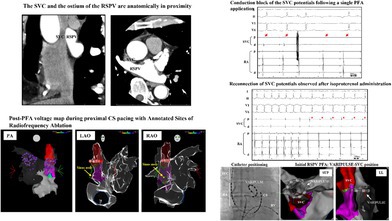

Durable pulmonary vein isolation (PVI) remains the cornerstone of catheter ablation for atrial fibrillation (AF) and can be achieved using radiofrequency energy, cryoballoon, hot balloon, or the novel pulsed field ablation (PFA). PFA utilizes ultrafast electrical pulses for nonthermal ablation, selectively and efficiently permeabilizing cardiac cells through irreversible electroporation and inducing targeted cell death.

However, delayed or isolated superior vena cava (SVC) conduction has been documented during the right superior pulmonary vein (RSPV) ablation using different modalities. The anatomical proximity between the RSPV and SVC has been correlated with delayed SVC potentials.^1^

Recently, PFA has been reported to potentially lead to SVC conduction delay and isolation following RSPV ablation.^2^

Herein, we report a case of transient conduction block of SVC potentials immediately following PFA of the RSPV.

A 61‐year‐old man with hypertension and paroxysmal AF was referred to our hospital for catheter ablation due to increasing AF episodes. Imaging and laboratory evaluations, including chest radiography, echocardiography, and blood tests, were unremarkable.

Preoperative contrast‐enhanced computed tomography revealed no thrombus in the left atrium (LA) and demonstrated extreme proximity between the RSPV and SVC (Figure 1, 0.3 mm longitudinally, 0.4 mm transversely).

**FIGURE 1:**
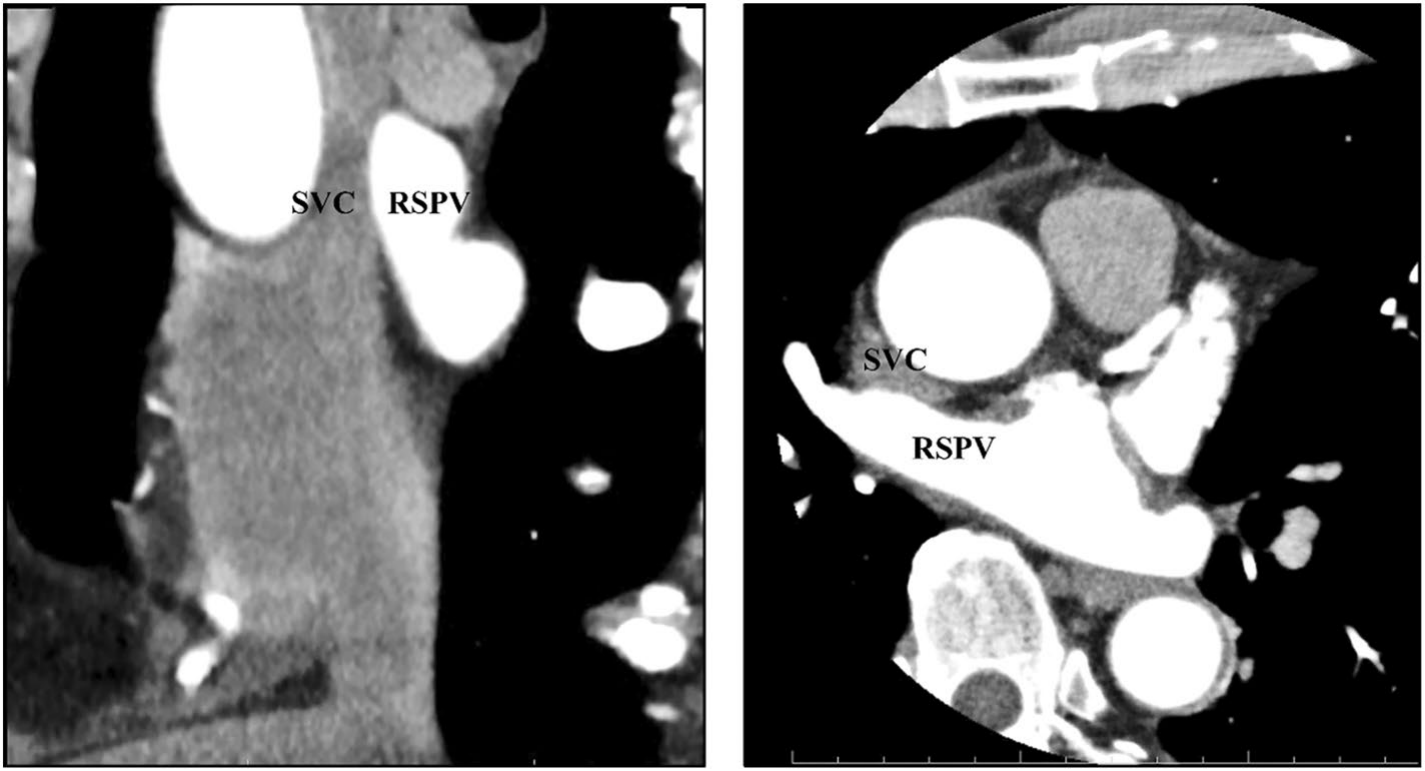
The superior vena cava (SVC) and the ostium of the right superior pulmonary vein (RSPV) were notably in close proximity. The anteroposterior diameter of the SVC was reduced to 6.40 mm because of compression by the aorta. The distance between the RSPV and SVC was 0.30 mm in the longitudinal view (left panel) and 0.40 mm in the transverse view (right panel).

The PFA procedure was performed under general anesthesia as follows. A 20‐electrode catheter (BeeAT; Japan Lifeline, Tokyo, Japan) was positioned in the coronary sinus (CS) along the lateral right atrial (RA) wall via the right internal jugular vein. An 8.5‐Fr Swartz long sheath and a steerable introducer sheath (VIZIGO; Biosense Webster Inc., Irvine, CA, USA) were introduced through the femoral vein. CARTOSOUND imaging via intracardiac echocardiography (CARTO‐Sound; Biosense Webster, Inc.) was used to map the RA and LA. Following transseptal puncture, a fast anatomical map was created during CS pacing using a multi‐electrode mapping catheter (OCTARAY Nav; Biosense Webster Inc.), guided by the CARTO3 three‐dimensional electroanatomical mapping system (Biosense Webster Inc.). PVI utilizing PFA was conducted with a CARTO3 system. Before PVI, a 20‐pole electrode catheter was positioned at the right ventricular apex to monitor potential vagal reflex activation. Initially, a VARIPULSE catheter (Biosense Webster, Inc.) was introduced into the RSPV, with the tissue proximity index (TPI) confirming contact. The 20‐electrode catheter placed in the CS‐RA was confirmed under fluoroscopic guidance, and the distal RA potential was anatomically identified as an SVC potential.

Before ablation, a potential was observed in the SVC. Following a single PFA application to the RSPV, the SVC potential was immediately eliminated (Figure 2). Subsequently, a total of 12 applications were administered to the RSPV, with additional applications at the carina site to address a conduction gap. RSPV isolation was confirmed by OCTARAY mapping. Ultimately, complete isolation was achieved after a total of 15 successful applications.

**FIGURE 2:**
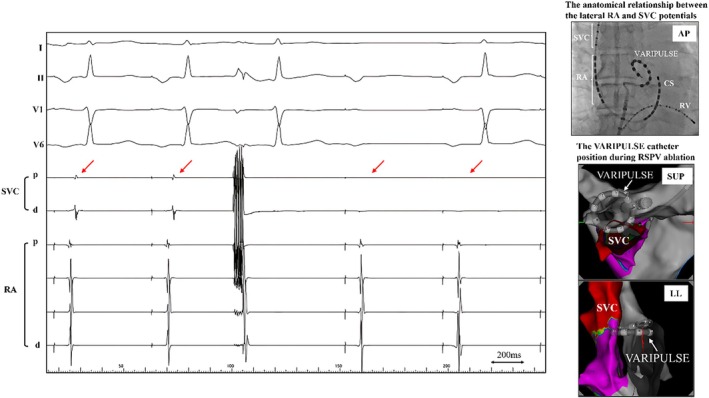
The right side of the upper panel shows a fluoroscopic image illustrating catheter positioning during pulmonary vein isolation (PVI). A 20‐electrode catheter was positioned via the jugular vein in the coronary sinus (CS) and along the lateral right atrial (RA) wall. In this image, the VARIPULSE catheter is located on the posterior wall of the left atrium (LA), while the distal end of the RA electrode, situated above the LA roof, is identified as representing the superior vena cava (SVC) potential. The right side of the middle and lower panels shows the positioning of the VARIPULSE catheter and SVC during the initial pulsed field ablation (PFA) application to the right superior pulmonary vein (RSPV). The left panel demonstrates the observation of SVC potentials before PFA, followed by the conduction block of the SVC potentials following a single PFA application (indicated by red arrows). AP, anteroposterior view; d, distal; LL, left lateral; p, proximal; RV, right ventricular; SUP, superior view.

PFA was subsequently administered to the remaining three PVIs, totaling 60 applications across the four pulmonary veins. However, isoproterenol (10 μg) administration to provoke non‐pulmonary vein foci and other tachyarrhythmias led to reconnection of SVC potentials within 2 min (Figure 3).

**FIGURE 3:**
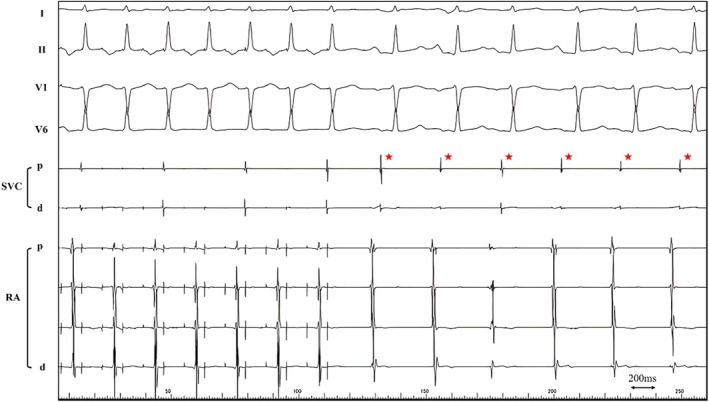
The panel shows the results of the tachycardia induction test following isoproterenol administration. Initially, the superior vena cava (SVC) potentials were dissociated but reconnected 2 min after administration (red star mark). d, distal; p, proximal; RA, right atrium.

In response to the emergence of extrasystoles originating from the SVC, SVC isolation was performed. SVC voltage mapping during CS pacing with the OCTARAY catheter after 15 PFA applications to the RSPV revealed a circumferential, extensive low‐voltage region (Figure 4). Subsequently, the anterior, septal, and posterior walls of the SVC were ablated using a QDOT catheter (Biosense Webster Inc.). Although the lateral wall was not ablated due to the risk of diaphragmatic nerve paralysis, SVC potentials were successfully eliminated, achieving complete isolation. No complications, including sinus node dysfunction or phrenic nerve paralysis, were observed following PFA or subsequent SVC ablation. The procedure was concluded after confirmation of the absence of potentials in both the pulmonary veins and SVC.

**FIGURE 4:**
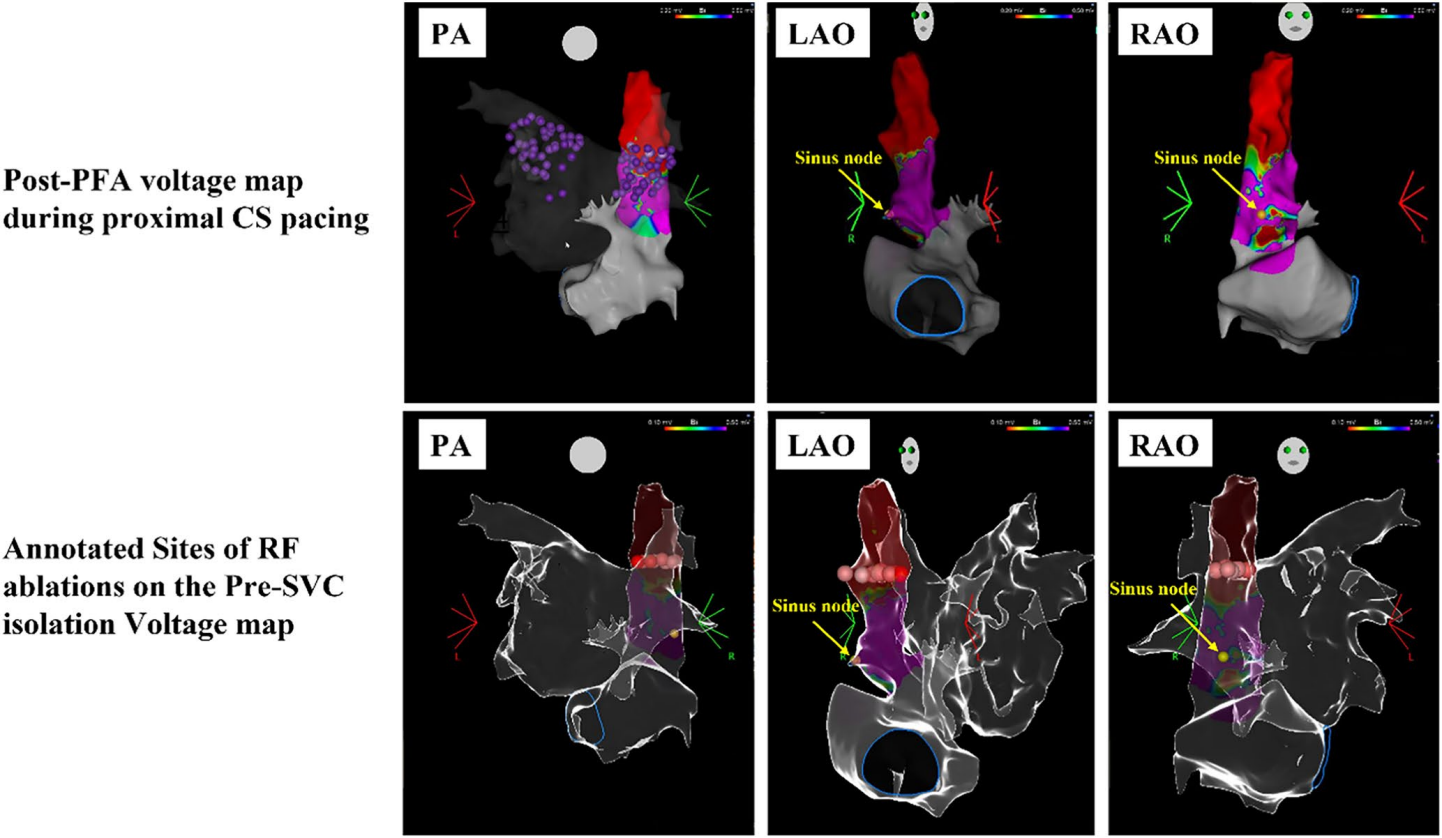
The upper panel shows post‐pulsed field ablation (PFA) voltage mapping during proximal coronary sinus (CS) pacing before superior vena cava (SVC) isolation. Purple tags indicate PFA applications. Low‐voltage areas are observed encompassing the entire circumference. The lower panel focuses on the annotation tags indicating the locations of radiofrequency (RF) ablations, superimposed on the voltage map acquired before SVC isolation. The sinus node, identified before SVC isolation, is indicated by the yellow tag in the middle and right images of both panels. SVC isolation was performed at the SVC–right atrial (RA) junction, corresponding anatomically to the roof of the left atrium. All radiofrequency applications were delivered with meticulous attention to maintaining an adequate safety margin from the sinus node. LAO, left oblique view; PA, posteroanterior view; RAO, right oblique view.

The patient was discharged 2 days post‐ablation without complications. During the 6‐month follow‐up period, without antiarrhythmic medication, the patient remained asymptomatic with no AF recurrence.

To our knowledge, this is the first report documenting immediate conduction block of SVC potentials following PFA, with subsequent reconnection. The anatomical proximity of the RSPV and SVC has previously been associated with delayed SVC conduction and isolation following PFA to the RSPV.^2^ However, unlike prior reports, conduction block of SVC potentials occurred immediately after PFA.

Notably, PFA applied with higher contact force or a positive TPI can create deeper and broader lesions.^3^, ^4^ In this case, the immediate SVC conduction block following a single PFA application was likely due to extensive lesions associated with a positive TPI. Additionally, the markedly short anteroposterior SVC diameter (6.4 mm) may have increased the effects of PFA, extending beyond the posterior septal interface near the RSPV and potentially involving the anterior wall, resulting in circumferential SVC isolation.

Furthermore, PFA lesions are characterized by a durable core with a transient peripheral zone, necessitating multiple applications per vein even after initial isolation. In this case, a total of 15 PFA applications were administered to the RSPV, achieving isolation; however, SVC potential conduction was subsequently restored, suggesting a transient effect on the SVC. This may be attributed to regression of borderline lesions at the periphery of the effective electric field, likely reversible in nature.^3^, ^4^ Consequently, SVC isolation during RSPV ablation with PFA may be transient, warranting additional ablation.

A limitation of this case study is the absence of pre‐ablation SVC voltage mapping, which would have facilitated evaluation of both the extent of PFA‐induced ablation and baseline SVC conduction characteristics before the procedure. It would also have definitively ruled out the initial absence of potential in the anterolateral wall, with conduction possibly confined to the posterior septum.

In cases where the RSPV and SVC are in proximity and the anteroposterior diameter of the SVC is reduced, the effects of PFA may extend beyond the posterior septum and involve the anterolateral wall, leading to SVC conduction block. However, lesions that do not directly involve the PFA tend to regress and are reversible, suggesting that SVC conduction block may be transient.

## FUNDING INFORMATION

This research received no specific grant from any funding agency in the public, commercial, or not‐for‐profit sectors.

## CONFLICT OF INTEREST STATEMENT

None of the authors has conflicts of interest to declare.

## ETHICS STATEMENT

All procedures were performed in accordance with the ethical standards of the institutional research committee and the 1964 Declaration of Helsinki and its later amendments or comparable ethical standards.

## CONSENT TO PARTICIPATE AND PUBLISH

Informed consent was obtained from the patient for participation and publication of this study.
